# miR-155 in the progression of lung fibrosis in systemic sclerosis

**DOI:** 10.1186/s13075-016-1054-6

**Published:** 2016-07-05

**Authors:** Romy B. Christmann, Alicia Wooten, Percival Sampaio-Barros, Claudia L. Borges, Carlos R. R. Carvalho, Ronaldo A. Kairalla, Carol Feghali-Bostwick, Jessica Ziemek, Yu Mei, Salma Goummih, Jiangning Tan, Diana Alvarez, Daniel J. Kass, Mauricio Rojas, Thiago Lemos de Mattos, Edwin Parra, Giuseppina Stifano, Vera L. Capelozzi, Robert W. Simms, Robert Lafyatis

**Affiliations:** Boston University School of Medicine, E501, Arthritis Center, Medical Campus, 72 East Concord Street, Boston, MA 02118-2526 USA; Hospital das Clinicas da Faculdade de Medicina da Universidade de São Paulo, São Paulo, SP Brazil; Universidade CEUMA, São Luís do Maranhão, MA Brazil; Medical University of South Carolina, Charleston, SC USA; University of Pittsburgh, Division of Pulmonary, Allergy, and Critical Care Medicine, and the Dorothy P. and Richard P. Simmons Center for Interstitial Lung Disease, Pittsburgh, PA USA; Universidade do Estado do Amazonas, Manaus, AM Brazil

**Keywords:** Systemic Sclerosis, Lung fibrosis, Gene expression, microRNA, Biomarker, miR-155, Bleomycin

## Abstract

**Background:**

MicroRNA (miRNA) control key elements of mRNA stability and likely contribute to the dysregulated lung gene expression observed in systemic sclerosis associated interstitial lung disease (SSc-ILD). We analyzed the miRNA gene expression of tissue and cells from patients with SSc-ILD. A chronic lung fibrotic murine model was used.

**Methods:**

RNA was isolated from lung tissue of 12 patients with SSc-ILD and 5 controls. High-resolution computed tomography (HRCT) was performed at baseline and 2–3 years after treatment. Lung fibroblasts and peripheral blood mononuclear cells (PBMC) were isolated from healthy controls and patients with SSc-ILD. miRNA and mRNA were analyzed by microarray, quantitative polymerase chain reaction, and/or Nanostring; pathway analysis was performed by DNA Intelligent Analysis (DIANA)-miRPath v2.0 software. Wild-type and miR-155 deficient (miR-155ko) mice were exposed to bleomycin.

**Results:**

Lung miRNA microarray data distinguished patients with SSc-ILD from healthy controls with 185 miRNA differentially expressed (*q* < 0.25). DIANA-miRPath revealed 57 Kyoto Encyclopedia of Genes and Genomes pathways related to the most dysregulated miRNA. miR-155 and miR-143 were strongly correlated with progression of the HRCT score. Lung fibroblasts only mildly expressed miR-155/miR-21 after several stimuli. miR-155 PBMC expression strongly correlated with lung function tests in SSc-ILD. miR-155ko mice developed milder lung fibrosis, survived longer, and weaker lung induction of several genes after bleomycin exposure compared to wild-type mice.

**Conclusions:**

miRNA are dysregulated in the lungs and PBMC of patients with SSc-ILD. Based on mRNA-miRNA interaction analysis and pathway tools, miRNA may play a role in the progression of the disease. Our findings suggest that targeting miR-155 might provide a novel therapeutic strategy for SSc-ILD.

**Electronic supplementary material:**

The online version of this article (doi:10.1186/s13075-016-1054-6) contains supplementary material, which is available to authorized users.

## Background

Systemic sclerosis (SSc) is a chronic autoimmune disease in which pulmonary involvement, particularly interstitial lung disease (SSc-ILD), is known to be the leading cause of mortality [1–3]. The prevalence of SSc-ILD is high and approximately 15–30 % of patients will progress to severe lung fibrosis. However, distinguishing those who will progress from those who will have slow or stable disease remains challenging [4, 5]. Using mRNA expression analysis of lung tissue in a prospective cohort of patients with SSc-ILD, we recently described immune activation pathways and upregulated transforming growth factor-β (TGFβ)-induced signature genes associated with progressive lung fibrosis [6].

MicroRNA (miRNA) are small RNA that are of vital importance in regulating gene expression within cells, having critical roles in innate and adaptive immunity in general [7]. In addition, there is increasing evidence they play a role in fibrosis and are strongly associated with the pathogenesis of a wide range of human diseases [8, 9]. miRNA are remarkably stable and reliable for detection in the blood and urine and bring new hope to the development of clinical biomarkers. More importantly, disease-associated miRNA represent a new class of therapeutic targets.

Using high-throughput technology, we show that many miRNA are dysregulated in the lungs of patients with SSc-ILD. In addition, selected miRNA are associated with progressive lung fibrosis based on image lung score and lung function tests, with miR-155 as one of the best markers. miR-155-deficient mice survive longer and develop milder lung fibrosis. Therefore, miR-155 could be a potential novel therapeutic target for lung fibrosis in patients with SSc.

## Methods

### Study participants

Between January 2002 and July 2004, 28 consecutive patients with SSc-ILD, identified on HRCT as having associated respiratory symptoms and/or reduced performance on a pulmonary function test (PFT) underwent an open lung biopsy and were followed for at least 3 years. Twelve subjects involved in the previous study [6] were selected (sixteen samples were not included due to lack of lung material for miRNA isolation). The patients included in the current study were classified as having diffuse SSc (dSSc) (n = 7) and limited SSc (lcSSc) (n = 5), according to diagnostic [10] and subtype criteria [11]. Controls were five samples of normal histological healthy lung tissue obtained during lung cancer surgical resection. Lung explants from controls and patients with SSc-ILD were obtained from lung transplants at the University of Pittsburgh Medical Center (healthy controls, n = 6; SSc-ILD, n = 6) and used for fibroblast culture. Peripheral blood mononuclear cells (PBMC) were collected from healthy controls (n = 6) and patients with SSc-ILD (n = 13) from Boston University Medical Center. Informed consent was obtained from all patients and healthy subjects involved in the study. The Boston University Medical Center, the University of Sao Paulo, and the University of Pittsburgh Institutional Review Boards reviewed and approved the conduct of this study.

### Histological diagnosis

Two pathologists (VLC and ERP) classified the lung specimens as previously described [6].

### High-resolution computed tomography

Two pulmonologists (CRC and RC) performed blinded evaluation of the images. As described previously [6] each lung was divided into three zones: upper (lung apex to aortic arch); middle (aortic arch to inferior pulmonary veins); and lower (inferior pulmonary veins to lung bases). For baseline and follow up HRCT, the extent of the pulmonary abnormality was scored as previously described [6]. The sum of all scores on the lung zones is referred to as FibMax. A delta FibMax, which is the difference between the follow-up FibMax and the baseline FibMax, was used to evaluate the progression of the lung fibrosis.

### Lung biopsy

Open lung biopsy was performed by formal thoracotomy, avoiding honeycombing areas [6].

### Follow up

**Fig. 1:**
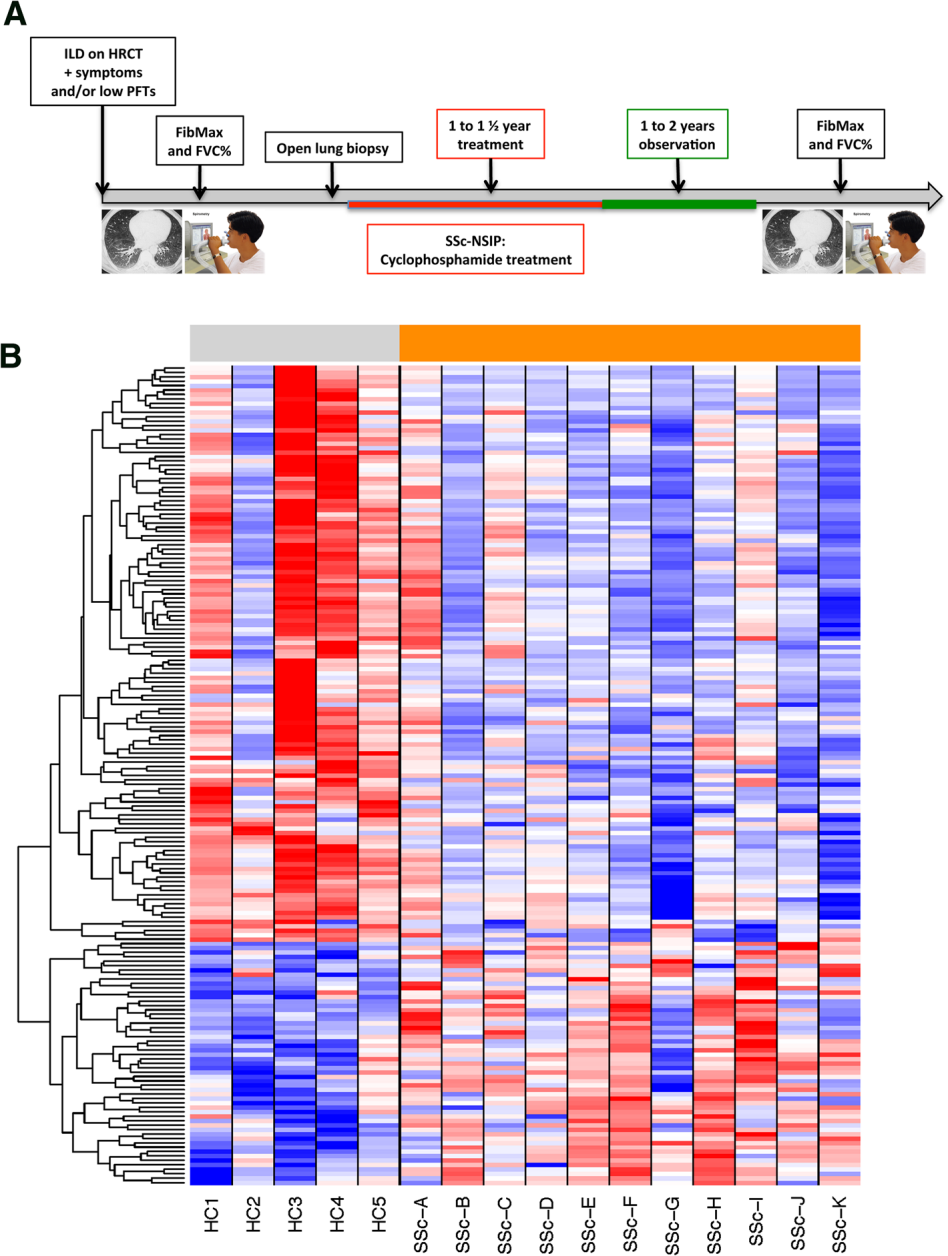
**a** Timeline of the study. See “Methods” for more details. **b** Heatmap of whole lung homogenate showing the expression of genes clustered using complete linkage hierarchical supervised clustering. *Gray* indicates healthy controls (*HC1* to *HC5*; n = 5); *orange* indicates patients with systemic sclerosis interstitial lung disease (SSc-ILD) (*SSc-A* to *SSc-K*; n = 12). Each miRNA was *z*-score-normalized across all samples and was scaled to *red* and *blue* (≥2 or ≤ –2, respectively) and *white* indicating a *z* score of zero. *ILD* interstitial lung disease, HRCT high-resolution computer tomography, *PFT* pulmonary function test, *FibMax* HRCT lung score, *FVC%* percent forced vital capacity, *NSIP* non-specific interstitial pneumonia

Patients with evidence of non-specific interstitial pneumonia (NSIP) on lung biopsy were treated with monthly intravenous cyclophosphamide (0.5–1.0 g/m^2^) for at least one year (see previous report for details [6]). HRCT and PFT were performed before biopsy and repeated 2–3 years after the one-year treatment (Fig. 1a).

### RNA and microRNA isolation and microarray analysis hybridization

Total RNA and miRNA from human lung biopsy tissue, lung fibroblasts, and PBMC were extracted using microRNeasy Mini Kits (Qiagen). RNA samples were stored at –80 °C.

### MicroRNA and messenger RNA quantitative PCR (qPCR)

Complementary DNA (cDNA) was transcribed using the TaqMan MicroRNA Reverse Transcription Kit (Life Technologies) and Reverse Transcriptase (Gibco BRL) and primers using 200 ng of total RNA and according to the manufacturer’s protocol. Samples were diluted 1:15 and followed the TaqMan Universal PCR Master Mix (Life Technologies) protocol. Specific miRNA primers were used for miR-155, miR-21, miR-182, miR-15b, miR-193a, and RNU6B (Life Technologies). Specific mRNA primers were used for collagen type 3 alpha-1 (Col3a1), MS4A4A, periostin (POSTN), and 18S (Life Technologies). miRNA and mRNA qPCR was performed using the TaqMan assay and StepOne Plus Real Time PCR system (Applied Biosystems) in a 20-ul reaction for 40 cycles. Gene expression was analyzed using the difference in cycle threshold (ΔCt) method. The Ct values of the miRNAs were normalized to RNU6B and of the mRNA were normalized to 18S as an internal control using the equation:

ΔCt = Ct reference – Ct target and expressed as ΔCt.

### Microarray data clustering

All procedures were performed at Boston University Microarray Resource Facility as described in the Affymetrix GeneChip 3′IVT Express user manual (Affymetrix, Santa Clara, CA, USA; www.affymetrix.com). For mRNA arrays biotin-labeled amplified total RNA was purified, fragmented, and hybridized to Affymetrix U133A 2.0 microarrays. The MAS5 algorithm with global scaling normalization was used to generate gene-level expression data. For miRNA arrays miRNA was purified, fragmented and hybridized to Affymetrix GenChip miRNA 3.0 microarrays. The signal of the samples was amplified and microarrays were immediately scanned using Affymetrix GeneArray Scanner 3000 7G Plus (Affymetrix, Santa Clara, CA, USA). The resulting CEL files were summarized using the Affymetrix Expression Console (current version 1.1). Clustering was performed using Cluster 3.0 software. Both mRNA and miRNA Affymetrix data [GEO:GSE81294] and SubSeries linked to GSE81294 [GEO:GSE81292; GEO:GSE81293] are available at the public repository Gene Expression Omnibus. Additional file 1: Table S1 and Additional file 2: Table S2 contain all the miRNA and mRNA, respectively, that were significantly different in patients with SSc-ILD and controls.

### Fibroblast culture

Lung tissue was minced and primary fibroblasts cultured using the outgrowth method as previously described [12]. Lung fibroblasts were cultured in DMEM supplemented with 10 % fetal bovine serum and penicillin/streptomycin and utilized at passages 2–4. Fibroblasts (100 % confluent) were incubated in serum-free DMEM overnight prior to stimulation with TGFß (R&D System; 2.5 ng/ml), recombinant human IL-13 (R&D Systems, 20 ng/ml), or interferon-alpha (IFN) (R&D Systems; 500 U/ml) for 18 hours. Total RNA from fibroblasts was transferred in Qiazol buffer and purified using the miRNease mini kit protocol (Qiagen). RNA samples were stored at –80 °C.

### Peripheral blood mononuclear cells

Blood was collected in cell preparation tubes (CPTs) designed for one-step cell separation (Becton Dickinson) from healthy controls and patients within 3 months of the date when the lung function tests were performed. The sample was immediately mixed and centrifuged at 1800 g at ambient temperature for 30 minutes. Total RNA from PBMC was extracted using the miRNease mini kit protocol (Qiagen). RNA samples were stored at –80 °C.

### MicroRNA Nanostring technology

RNA (100 ng from each sample) was used for the miRNA analysis. The miRNA library contains 800 of the most relevant miRNA described in the literature. Normalization was performed using the average of expression of the top 20 most-expressed miRNA in all samples. The minimal threshold for the detection of miRNA was considered as 50 counts.

### RNA Nanostring technology

RNA (100 ng of from each mouse) was used for RNA analysis. The lung gene panel was built based on a microarray analysis of the lungs of mice exposed to a bleomycin-pump compared to PBS in previous experiments (unpublished results). Normalization was performed using nine housekeeping genes that were not affected by the bleomycin-pump model.

DNA Intelligent Analysis (DIANA) DIANA-miRPath v2.0 utilizes miRNA targets based on DIANA-microT-CDS, which predicts miRNA-gene interaction including the binding region, position, and type, and/or miRNA-gene interaction experimentally validated from Tarbase v7.0 [13]. Combined analysis results for miRNA and pathway-related information was obtained from miRBase 18 [14] and the Kyoto Encyclopedia of Genes and Genomes (KEGG) v58 [15]. The selected top 30 upregulated and downregulated miRNA were used (Additional file 1: Table S1).

### Bleomycin lung fibrosis model

C57BL/6 J (B6) and B6.Cg-Mir155^tm1.Rsky/^J mice were purchased from The Jackson Laboratory (Bar Harbor, ME, USA). Age-matched and sex-matched mice were used in the experiments at 8 to 10 weeks. Mice were kept in pathogen-free conditions with food and water. The in vivo study was approved by the Animal Ethics Committee at the Boston University School of Medicine. Mice were chronically exposed to PBS or Bleomycin (90 U/Kg) by subcutaneous osmotic pump injections for 7 days. Mice were observed and sacrificed on day 28. The survival curve was calculated. Lungs were harvested for histology and gene expression. H&E and Masson’s Trichrome staining were performed. The Ashcroft lung score was measured blinded by one investigator (RBC).

### Statistical analysis

Data analysis was performed using GraphPad Prism 6 software, version 6.0a (GraphPad Software, San Diego, CA, USA). Multiple group comparisons were analyzed by one-way analysis of variance (ANOVA) with Bonferroni adjustment for multiple comparisons. Two-group comparisons were analyzed by Student’s *t* test or the Mann-Whitney test. Correlation was tested and presented as Pearson’s correlation coefficient (*r*). The survival fraction was calculated by the Kaplan-Meier test with 95 % confidence intervals computed by the asymmetrical method using the log-rank test.

## Results

### Baseline characteristics of patients with systemic sclerosis interstitial lung disease

A timeline of the study is summarized in Fig. 1a. We have previously described all the demographic and FibMax data of these patients with SSc-ILD before and after treatment [6]. Note that despite treatment, most patients with SSc-ILD developed progressive disease based on the FibMax, with a mean baseline score of 6.88 ± 4.47 compared to 12.76 ± 6.59 after treatment; *p* = 0.006. The delta FibMax was used to gauge the progression of lung fibrosis as previously described [6]. Healthy control lungs were obtained from cancer-free resected lungs.

### Patients with systemic sclerosis interstitial lung disease have distinct microRNA gene expression patterns

We analyzed lung tissue samples from healthy controls (n = 5) and patients with SSc-ILD (n = 12) using miRNA microarrays. Both middle and lower lobe samples were analyzed in five of the patients with SSc-ILD, while only the lower lobes were analyzed in seven patients. One patient was excluded from the miRNA analysis on the basis of poor quality metrics. To identify genes with differential expression associated with SSc-ILD, Student’s two-sample *t* test was performed to compare SSc-ILD (expression averaged for each gene from both lung biopsy sites) and control samples (*p* < 0.05). A false discovery rate (FDR)-corrected *p* value (*q* < 0.25) was computed after removing miRNA present in ≤25 % of the samples, to eliminate miRNA with low overall expression. A heatmap of the most informative miRNA (n = 185 genes) was created containing the miRNA with the most significant differential expression between SSc-ILD and control samples. Most of the miRNA were downregulated in patients compared to the healthy controls (Fig. 1b). The complete list of significantly different miRNA can be found in Additional file 1: Table S1.

**Fig. 2:**
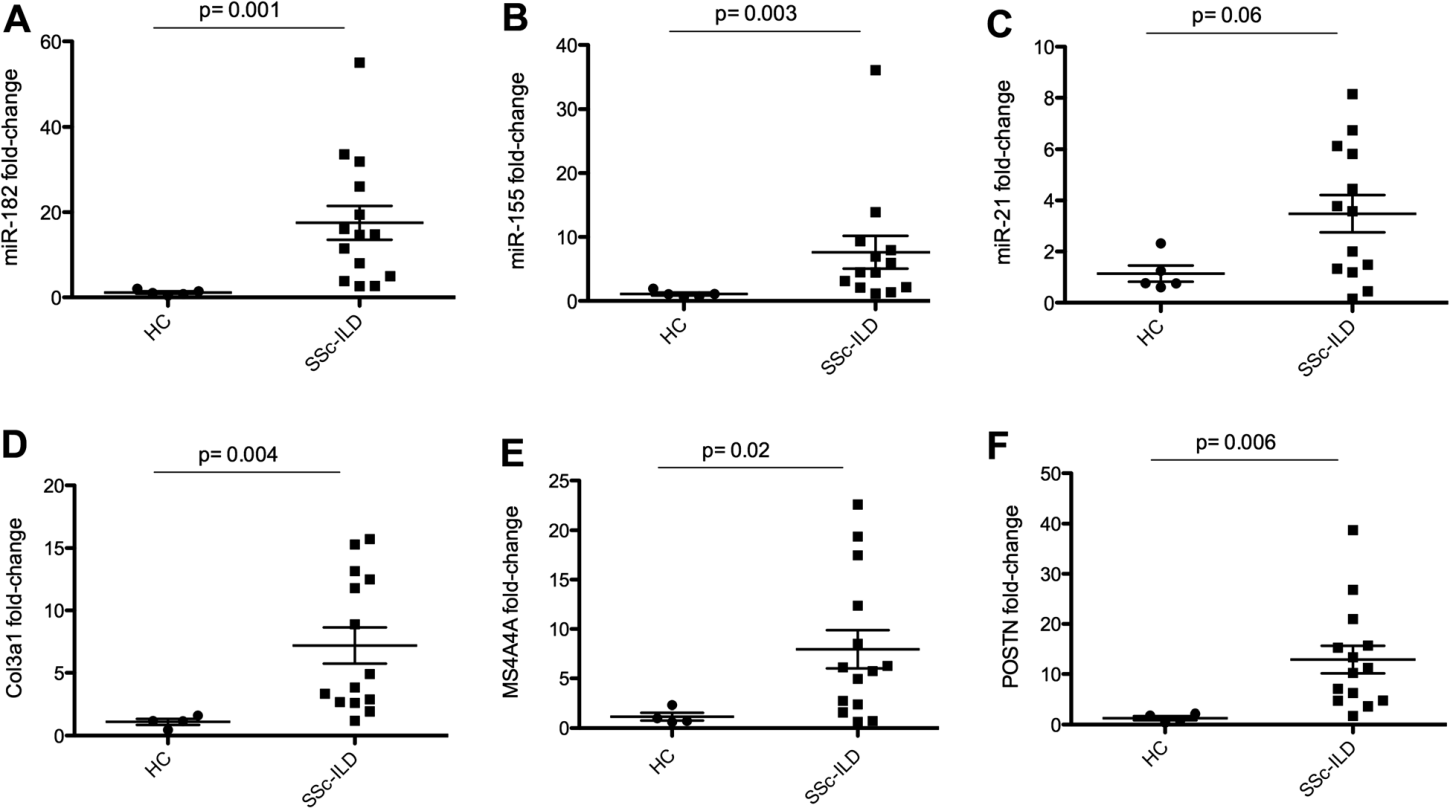
Whole lung homogenate with miRNA and mRNA expression by qPCR of miR-182 (**a**), miR-155 (**b**), miR-21 (**c**), collagen type 3 alpha-1 (*Col3a1*) (**d**), MS4A4A (**e**), and periostin (*POSTN*) (**f**) in healthy controls (*HC*) (n = 5) and patients with systemic sclerosis interstitial lung disease (*SSc-ILD*) (n = 14). Data are expressed as fold-change normalized to miRNA (RNU6B) or mRNA (18S) average expression in HC samples. Student’s *t* test; *p* < 0.05 was considered significant

We validated several miRNA that were upregulated in patients with SSc-ILD compared to the controls, by qPCR. miR-182 was highly expressed in the lung tissue from patients with SSc-ILD (mean fold-change 17.5 ± 14.8) compared to controls (mean fold-change 1.1 ± 0.6; *p* = 0.001. Fig. 2a). miR-155 was upregulated in patients with SSc-ILD (mean fold-change 7.6 ± 9.2) compared to controls (mean fold-change 1.0 ± 0.5; *p* = 0.003. Fig. 2b), and miR-21 was upregulated in patients with SSc-ILD (mean fold-change 3.4 ± 2.6) compared to controls (mean fold-change 1.1 ± 0.7; *p* = 0.06), although not statistically significant (Fig. 2c).

### Validation of relevant gene signatures in systemic sclerosis interstitial lung disease

In order to better understand the potential mRNA targeted by altered lung miRNA expression, we analyzed all samples for mRNA expression by microarray. We identified genes with differential expression associated with SSc-ILD, using Student’s two-sample *t* test (*p* < 0.05) corrected by FDR (*q* < 0.25). These microarray data confirmed the altered expression of hundreds of genes in SSc-ILD that we reported previously [6], such as chemokine C-C motif ligand 18 (CCL18), several collagens (types I and III), macrophage activation markers (for example, CD163 and MS4A4A), secreted phosphoprotein 1/osteopontin (SPP1), and others (Additional file 2: Table S2). Using qPCR, we validated several of the top upregulated genes in lung tissue from patients with SSc-ILD, such as COL3a1 (*p* = 0.004), MS4A4A (*p* = 0.02), and POSTN (*p* = 0.006) Fig. 2d-f.

### Messenger RNA and microRNA: a complex network

Taking advantage of our simultaneous analysis of mRNA and miRNA in the same cohort of patients with SSc-ILD and healthy controls we further investigated interactions between mRNA and miRNA lung expression. Pearson’s correlation was tested between selected upregulated and downregulated miRNA (see Additional file 1: Table S1) and the most highly upregulated and downregulated mRNA in SSc-ILD (see Additional file 2: Table S2) by microarray analysis, based on average fold-change gene expression compared to controls. miR-155, miR-21, and miR-4459 were the miRNA that were most strongly correlated and their interactions are shown in Table 1.

**Table 1:** Strongest positive and negative correlation between the most expressed messenger RNA (top upregulated and downregulated genes) and miR-155/miR-21/miR-4459 lung expression

Abbreviation	Gene name	Fold-change	*r* (miR-155)	*r* (miR-21)	r (miR-4459)
MMP1	Matrix metallopeptidase 1 (interstitial collagenase)	14.1	0.13	0.24	**-0.52**
MMP7	Matrix metallopeptidase 7 (matrilysin, uterine)	12.0	**0.57**	**0.54**	**-0.85**
KRT15	Keratin 15	7.1	0.27	0.40	**-0.51**
S100A2	S100 calcium binding protein A2	6.6	0.29	0.45	**-0.57**
PDIA3	Protein disulfide isomerase family A, member 3	6.2	**0.67**	**0.52**	**-0.76**
CDH3	Cadherin 3, type 1, P-cadherin (placental)	5.6	**0.60**	**0.60**	**-0.82**
IL13RA2	Interleukin 13 receptor, alpha 2	5.6	**0.75**	**0.81**	**-0.78**
IGHM	Immunoglobulin heavy constant mu	5.5	**0.67**	**0.52**	**-0.63**
SPP1	Secreted phosphoprotein 1	5.1	**0.69**	**0.55**	**-0.74**
GPR87	G protein-coupled receptor 87	4.7	0.29	0.32	-0.45
RRM2	Ribonucleotide reductase M2	4.7	**0.72**	**0.59**	**-0.57**
CTSE	Cathepsin E	4.4	**0.66**	**0.66**	**-0.91**
AADAC	Arylacetamide deacetylase	4.4	**0.60**	0.48	**-0.76**
CXCL6	Chemokine (C-X-C motif) ligand 6 (granulocyte chemotactic protein 2)	4.3	0.34	0.34	-0.30
CR2	Complement component (3d/Epstein Barr virus) receptor 2	4.2	**0.61**	0.37	-0.42
TYMS	Thymidylate synthetase	4.2	**0.72**	**0.58**	**-0.75**
CXCL13	Chemokine (C-X-C motif) ligand 13	4.1	**0.77**	**0.59**	-0.48
SCG5	Secretogranin V (7B2 protein)	4.1	**0.76**	**0.70**	**-0.70**
MEOX1	Mesenchyme homeobox 1	3.9	**0.59**	**0.52**	**-0.51**
TOP2A	Topoisomerase (DNA) II alpha 170 kDa	3.7	**0.68**	**0.62**	**-0.64**
POSTN	Periostin, osteoblast specific factor	3.7	**0.74**	**0.70**	**-0.74**
	Pearson’s correlation >0.5 or < -0.5	total	n = 16	n = 14	n = 17
IL6	Interleukin 6 (interferon, beta 2)	-45.2	**-0.50**	-0.44	**0.54**
SERPINE1	Plasminogen activator inhibitor type 1, member 1	-21.9	-0.45	-0.36	**0.60**
FOSB	FBJ murine osteosarcoma viral oncogene homolog B	-18.4	**-0.69**	**-0.66**	**0.73**
MT1M	Metallothionein 1 M	-15.8	**-0.50**	-0.41	**0.54**
DDX3Y	DEAD (Asp-Glu-Ala-Asp) box polypeptide 3, Y-linked	-14.3	-0.17	**-0.51**	0.33
RND1	Rho family GTPase 1	-13.6	-0.46	-0.37	0.40
IL8	Interleukin 8	-12.8	-0.40	-0.26	0.37
NXF3	Nuclear RNA export factor 3	-12.0	**-0.80**	**-0.75**	**0.89**
CSF3	Colony stimulating factor 3 (granulocyte)	-11.8	-0.50	-0.34	0.48
CCL2	Chemokine (C-C motif) ligand 2	-11.6	-0.47	-0.36	**0.54**
PTX3	Pentraxin 3, long	-11.4	-0.15	-0.02	0.27
IL12A	Interleukin 12A	-11.1	**-0.70**	**-0.64**	**0.85**
CCL20	Chemokine (C-C motif) ligand 20	-9.5	-0.25	-0.17	0.15
GADD45B	Growth arrest and DNA-damage-inducible, beta	-9.5	**-0.50**	-0.42	**0.54**
ESM1	Endothelial cell-specific molecule 1	-8.6	**-0.52**	-0.36	0.48
CXCL2	Chemokine (C-X-C motif) ligand 2	-8.5	-0.40	-0.44	0.46
SLC6A4	Solute carrier family 6 (neurotransmitter transporter, serotonin), member 4	-8.4	**-0.82**	**-0.87**	**0.72**
SLCO4A1	Solute carrier organic anion transporter family, member 4A1	-8.3	**-0.62**	**-0.50**	**0.64**
KDM5D	Lysine (K)-specific demethylase 5D	-8.2	-0.15	-0.48	0.37
S100A12	S100 calcium binding protein A12	-8.2	-0.45	-0.33	**0.53**
HAS2	Hyaluronan synthase 2	-8.2	-0.29	-0.27	0.30
	Pearson’s correlation >0.5 or < –0.5	total	n = 9	n = 6	n = 10

In sequence: gene symbol, gene name, average fold-change expression in SSc-ILD compared to healthy controls, *r* values for correlation between each gene and specific miRNA

### RNA and microRNA: pathway analysis

We selected the top upregulated and downregulated miRNA in patients with SSc-ILD compared to the controls (see Additional file 1: Table S1) for pathway analysis. First, we applied the DIANA-Tarbase v7.0 database in order to identify miRNA target genes experimentally validated in the literature (see “Methods”). Only a few miRNA from the selected group had validated target genes: miR-155, miR-21, miR-125a, miR-31, miR-205, miR-20a, let-7f, miR-182, let-7e, miR-199b, miR-199a, miR-663, and miR-193a. miR-155 (n = 812) and miR-21 (n = 581) had the largest list of target genes already validated.

To gain further insight into the function of the top dysregulated miRNA and their targets, DIANA-microT-CDS miRPath was applied to identify the significant KEGG pathways (see “Methods”): 57 KEGG biological processes were significantly enriched (*p* < 0.05, FDR-corrected) in SSc-ILD (Additional file 3: Figure S1). The 10 most highly implicated processes identified using this approach, with their related miRNA, can be found in Table 2.

**Table 2:** Top 10 Kyoto Encyclopedia of Genes and Genomes (KEGG) pathways related to the most expressed microRNA in the lungs of patients with systemic sclerosis interstitial lung disease compared to controls

KEGG pathway	*p* value	#genes	miRNA					
ECM-receptor interaction (hsa04512)	<1e-16	16	3	let-7e,	miR-4459,	let-7f		
Ubiquitin mediated proteolysis (hsa04120)	<1e-16	76	9	miR-182,	miR-141,	miR-125a,	miR-379,	miR-4484,
				miR-574,	miR-3613,	miR-4739,	miR-20a	
Wnt signaling pathway (hsa04310)	<1e-16	79	10	miR-155,	miR-205,	miR-1224,	let-7e,	miR-4459,
				miR-762,	miR-4668,	let-7f,	miR-3613,	miR-20a
MAPK signaling pathway (hsa04010)	<1e-16	132	13	miR-21,	miR-155,	miR-125a,	let-73,	miR-4530,
				miR-1207,	miR-149,	miR-762,	let-7f,	miR-199a,
				miR-199b,	miR-3613,	miR-20a		
Pathways in cancer (hsa05200)	<1e-16	166	15	miR-21,	miR-155,	miR-182,	miR-205,	miR-1224,
				let-7e,	miR-4459,	miR-4484,	let-7f,	miR-199a,
				miR-574,	miR-199b,	miR-3613,	miR-4739,	miR-20a
Axon guidance (hsa04360)	7.77E-16	72	11	miR-155,	miR-205,	miR-1224,	miR-379,	miR-4741,
				miR-762,	miR-4689,	miR-4484,	miR-3613,	miR-4739,
				miR-20a				
PI3K-Akt signaling pathway (hsa04151)	2.74E-14	137	12	miR-155,	miR-182,	let-7e,	miR-379,	miR-4459,
				miR-149,	miR-4668,	let-7f,	miR-199a,	miR-199b,
				miR-3613,	miR-20a			
TGF-beta signaling pathway (hsa04350)	5.88E-14	41	10	miR-21,	miR-205,	let-7e,	miR-4668,	miR-451a,
				miR-4484,	let-7f,	miR-3613,	miR-20a,	miR-455
ErB signaling pathway (hsa04012)	1.17E-13	40	12	miR-155,	miR-205,	miR-193a,	miR-200b,	miR-4530,
				miR-451a,	miR-4484,	miR-199a,	miR-199b,	miR-200c,
				miR-3613,	miR-20a			
Transcriptional misregulation in cancer (hsa05202)	1.23E-13	83	11	miR-182,	miR-205,	miR-1224,	let-7e,	miR-1207,
				miR-149,	miR-4668,	let-7f,	miR-3613,	miR-20a,
				miR-455				

In sequence: pathway names, significance levels based on *p* value, number of targeted genes, number of miRNA targeting each pathway, and list of miRNA related to each pathway

### miR-155 lung expression correlates with progressive systemic sclerosis interstitial lung disease

**Fig. 3:**
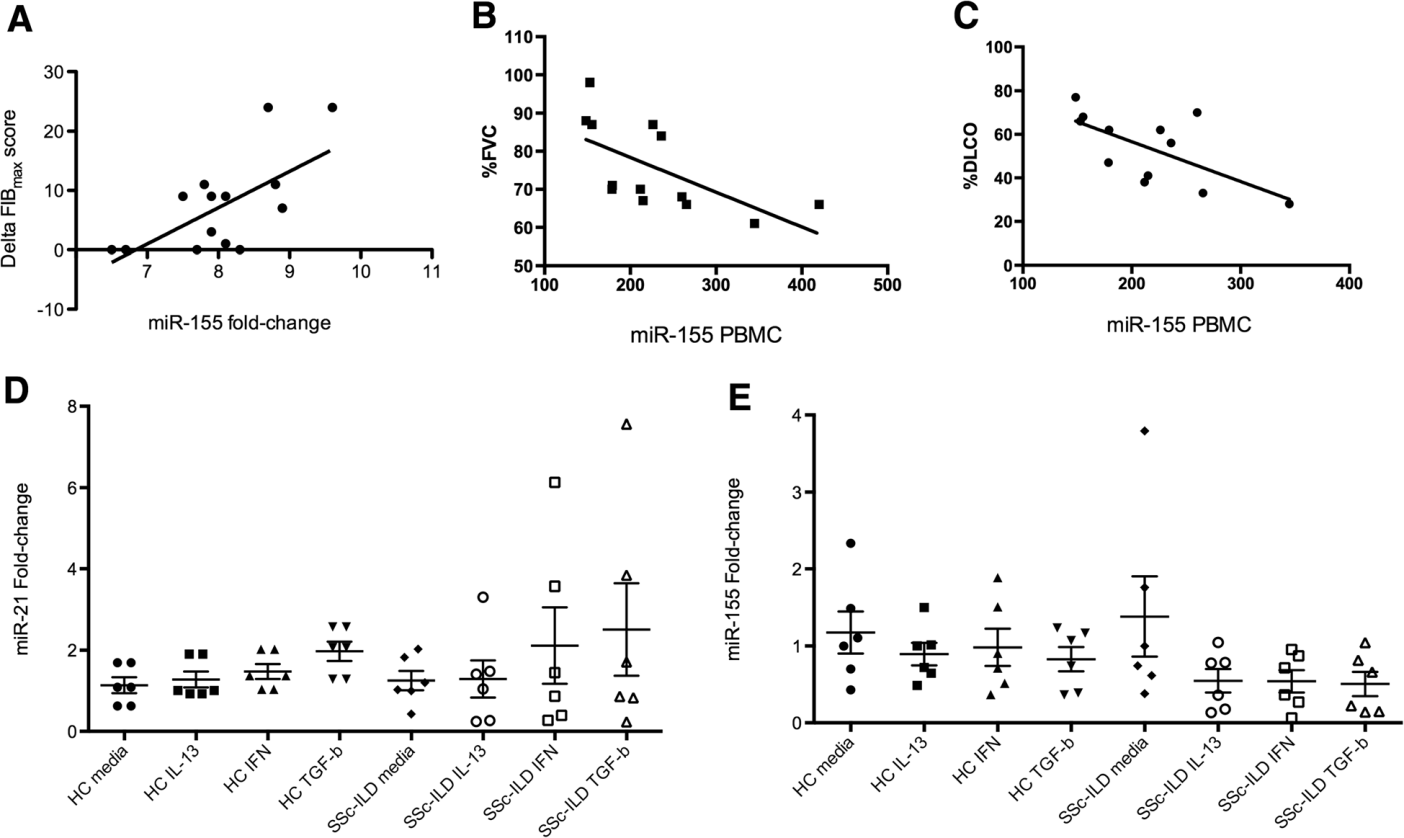
**a** Correlation between the delta high-resolution computerized tomography lung score (*Fib*_*Max*_) and mir-155 whole lung homogenate gene expression (*r* = 0.65, *p* < 0.001). **b** Correlation between miR-155 expression on peripheral blood mononuclear cells by Nanostring and percentage forced vital capacity (*%FVC*) (*r* = –0.60, *p* = 0.01), and **c** percentage diffusing capacity of the lung for carbon monoxide (*%DLCO*) (*r* = –0.58, *p* = 0.02). **d** miR-21 and **e** miR-155 expression on lung fibroblasts from cell lines of healthy controls (*HC*) and patients with systemic sclerosis interstitial lung disease (*SSc-ILD*) stimulated for 18 hours with media, IL-13, transforming growth factor-beta (*TGF-β*), and interferon-alpha (*IFN-α*) (see “Methods”) (analysis of variance, *p* > 0.05 for both miRNA). Data are expressed as fold-change compared to HC samples or media control on **a**, **b**, **d**, and **e**. Data are expressed as counts of gene expression and percentage on **b** and **c**. *p* < 0.05 was considered significant

There is an urgent need for a biomarker that predicts the progression of lung fibrosis in patients with SSc. miRNA are known to regulate a complex network; therefore, we tested the correlation between miRNA microarray lung gene expression and changes in the lung fibrosis score FibMax, the sum of all fibrotic scores in the six lung zones (see “Methods”). The delta FibMax of each patient, when available, was correlated with corresponding patient lung miRNA microarray fold-change gene expression (see Additional file 1: Table S1). Surprisingly, only four miRNA correlated positively with the delta FibMax (*r* > 0.4) with miR-155 having the strongest correlation (*r* = 0.65, *p* < 0.001, Fig. 3a), followed by miR-182 (*r* = 0.49, *p* = 0.06), miR-27a (*r* = 0.49, p = 0.06), and miR-21 (*r* = 0.47, *p* = 0.07). Five miRNA correlated negatively with the delta FibMax, with miR-143 having the strongest negative correlation (*r* = –0.64, *p* < 0.01), followed by miR-4270 (*r* = –0.59, *p* = 0.01), miR-4530 (*r* = –0.54, *p* = 0.03), miR-19b (*r* = –0.51, *p* = 0.05), and miR-4459 (*r* = –0.50, *p* = 0.05).

### miR-155 expression by peripheral blood mononuclear cells is correlated with progressive systemic sclerosis interstitial lung disease

PBMC gene expression has been used as a surrogate for rarely available lung tissue to further investigate lung complications in SSc, such as SSc-ILD and SSc pulmonary arterial hypertension (SSc-PAH) [16]. Therefore, we analyzed miRNA expression in SSc-ILD (n = 13) and control PBMC (n = 5) by Nanostring; demographic features are shown in Additional file 4: Table S3. Among the miRNA that were upregulated in the lungs of patients with SSc-ILD (Additional file 1: Table S1) let-7d (*p* = 0.01), miR-15b (*p* = 0.05), and miR-21 (*p* = 0.1) were the most expressed in PBMC from SSc-ILD compared to controls (Additional file 5: Table S4) based on fold-change expression.

More importantly, we observed that miR-155 expression on SSc-ILD PBMC was strongly negatively correlated with both %FVC (*r* = –0.60; *p* = 0.01) and %DLCO (*r* = –0.58; p = 0.02). let-7d was also strongly negatively correlated with %FVC (*r* = –0.65; *p* = 0.007) and with %DLCO (*r* = –0.56; *p* = 0.02). miR-21 PBMC expression did not correlate with lung function tests (%FVC *r* = 0.14; %DLCO *r* = 0.18; both *p* > 0.05).

### Activated lung fibroblasts have minor influence on the microRNA signature in systemic sclerosis interstitial lung disease

In order to elucidate whether fibroblasts might be responsible for the miRNA expression in SSc-ILD we stimulated healthy lung fibroblasts (n = 6) and fibroblasts from the lungs of patients with SSc-ILD (n = 6) with cytokines known to be involved in the progression of lung disease: TGFβ, IL-13, and IFN [6]. Fibroblasts were stimulated for 18 hours and miRNA expression was analyzed by Nanostring technology (Additional file 6: Table S5) and confirmed in a larger number of samples by qPCR (see “Methods”).

Expression of miR-21 was in general slightly increased after IFN and TGFβ stimulation of fibroblasts in SSc-ILD, although not statistically significant (Fig. 3d, *p* > 0.05). On the other hand, miR-155 expression was reduced overall after all three stimuli of fibroblasts in SSc-ILD, although not statistically significant (Fig. 3e, *p* > 0.05). There was mild expression of miR-193a and miR-15b in lung fibroblasts in both groups after all three stimuli (Additional file 7: Figure S2A and B, respectively).

### The absence of miR-155 protects mice from bleomycin-induced lung fibrosis

**Fig. 4:**
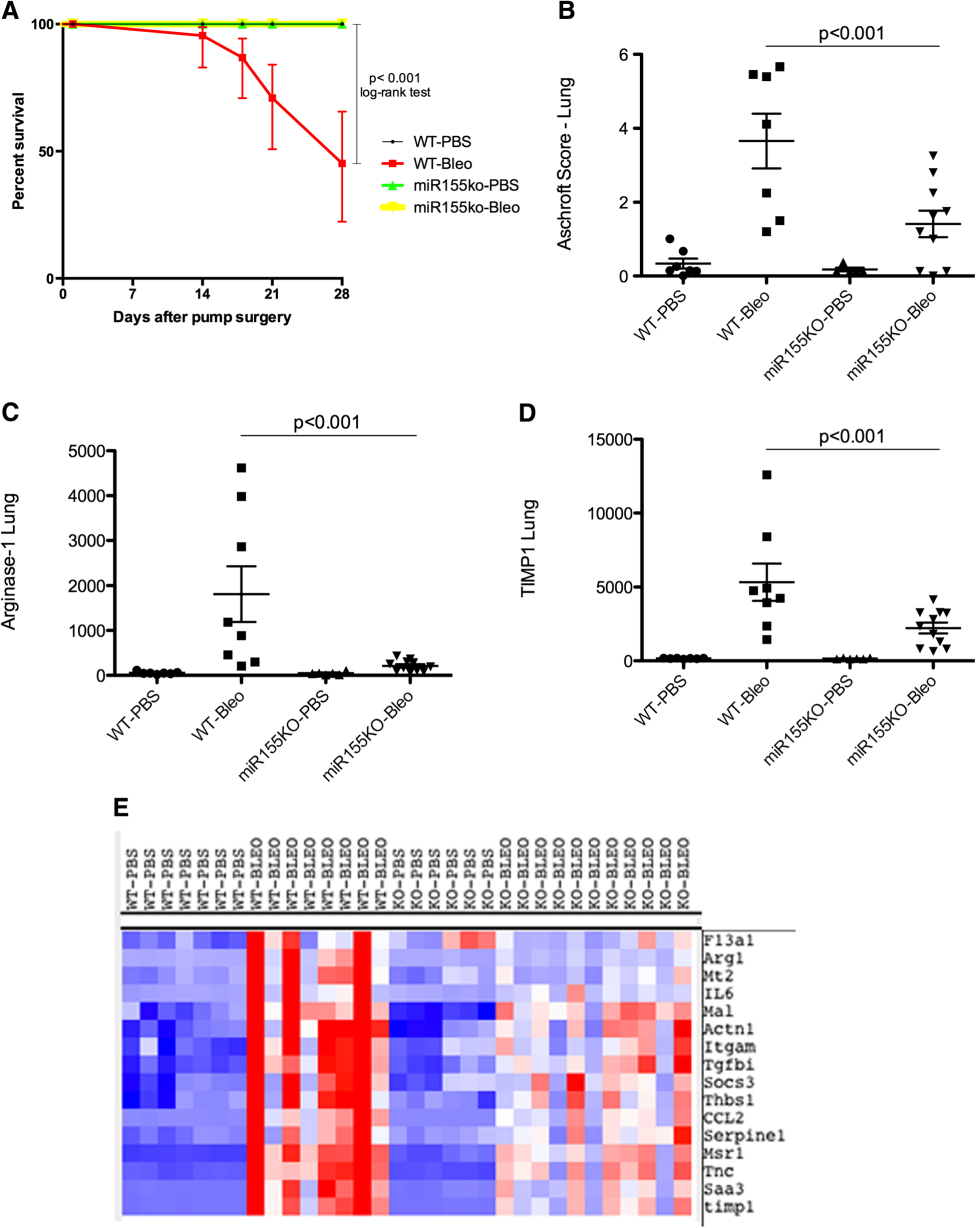
Milder lung fibrosis in miR-155 knockout (*KO*) mice. **a** Percent survival rate by Kaplan-Meier, log-rank test. **b** Ashcroft lung score assessed by Masson’s trichrome staining. Lung gene expression by Nanostring of Arginase-1 (**c**) and tissue inhibitor of metalloproteinase-1 (*TIMP1*) (**d**). **e** Cluster of the most affected genes by the absence of miR-155 (*blue*). Data are expressed by counting gene expression on **c** and **d**. Each microRNA was *z*-score-normalized across all samples and scaled to *red* and *blue* (≥2 or ≤ –2, respectively) and *white* indicating a *z* score of zero. Analysis of variance; *p* < 0.05 was considered significant. *WT* wild-type, *Bleo* bleomycin

We observed that miR-155 expression in the lungs and in the blood of patients with SSc-ILD are strongly correlated with progressive lung disease identified by image and by lung function tests, respectively. Therefore, we tested the bleomycin model of chronic lung fibrosis in mice deficient in miR-155 (miR-155 knockout (KO)) compared to wild-type mice (WT). miR-155 KO mice had a 100 % survival rate over the course of 28 days after starting bleomycin compared to a 45 % survival rate in WT mice; *p* < 0.001, log-rank test (Fig. 4a). WT (n = 7) and miR-155 KO (n = 6) mice exposed to PBS were used as controls. WT (n = 11) and miR-155 KO (n = 11) mice were exposed to bleomycin and three WT mice were found dead on days 8, 13, and 17, respectively. One WT mouse exposed to bleomycin was killed on day 20 due to poor conditions and was included in the lung Nanostring analysis. miR-155 KO mice (n = 11) developed milder fibrosis in the lungs as measured by the Ashcroft lung score (Fig. 4b, *p* < 0.001) compared to the WT (n = 7) exposed to bleomycin on day 28.

We tested the effect of miR-155 deletion on bleomycin-induced gene expression using a custom-designed Nanostring that we developed to detect proinflammatory and profibrotic genes upregulated in the lungs of bleomycin-treated lung tissue and lung tissue from patients with SSc-ILD. Overall, bleomycin-induced lung gene expression was lower in miR-155 KO mice (Additional file 8: Figure S3). Arginase-1 lung gene expression was strongly induced in the WT mice and correlated positively with the Ashcroft score (*r* = 0.70, *p* < 0.001). Arginase-1, a marker of M2 macrophages (Fig. 4c), and tissue inhibitor of metalloproteinase-1 (TIMP1) lung expression (Fig. 4d) were induced significantly less after bleomycin in miR155 KO mice compared to WT mice. Expression of other bleomycin-induced genes was blunted in miR-155 KO lungs compared to WT mice and clustered with Arginase-1 and TIMP1 (Fig. 4e). On the other hand, expression of CD68, a general marker of macrophages, was induced similarly in both groups exposed to bleomycin (*p* > 0.05).

## Discussion

Dysregulation of the immune system is critical for the development of skin and lung involvement in SSc. In this regard, we have recently shown that this inappropriate inflammatory response in the lungs is strongly correlated with progressive lung fibrosis [6], along with a profibrotic TGFβ signature. miRNA are a new class of regulators that tightly control the overall inflammatory response. We show here that miRNA are intensely dysregulated in the lungs and in PBMC in patients with SSc-ILD. Moreover, we found that dysregulated gene expression of only a few miRNA, including miR-155, are strongly associated with progressive lung disease and that the absence of miR-155 is protective for the bleomycin-lung fibrotic model.

miR-155 controls differentiation of CD4+ T cells into its several subtypes to regulate B-cell differentiation and antibody production, and is highly expressed in activated monocytes/macrophages [17]. miR-155 is upregulated in several autoimmune disorders, such as rheumatoid arthritis [18], multiple sclerosis [19], systemic lupus erythematosus [20, 21], and ulcerative colitis [22]. In our cohort, we showed that miR-155 expression correlates highly with profibrotic gene expression, such as SPP1 and POSTN. We also observed strong correlation between miR-155 PBMC expression in patients with SSc-ILD and lung function tests. On the other hand, lung fibroblasts seem not to be the most relevant cell type driving the strong expression of miR-155 in the lungs in patients with SSc-ILD.

We showed the functional relevance of miR-155 in lung fibrotic disease where miR-155 KO mice survived longer and developed substantially less aggressive lung fibrosis based on lung phenotype and on a comprehensive lung gene expression analysis. This protective effect might be underestimated as three mice in the WT bleomycin group did not survive until day 28 and neither the lung score nor the lung gene expression were considered in the analysis. In addition, the absence of miR-155 in the model blocked the alternative activation of lung macrophages, which correlates strongly with progressive lung fibrosis in patients with SSc-ILD [6] and with the Ashcroft lung fibrotic score in our murine model. Yan et al. [23] recently showed protection of bleomycin cutaneous fibrosis in miR-155-deficient mice and reduced skin thickness after topical antagomiR-155 treatment in mice primed with subcutaneous bleomycin [23]. In the intratracheal bleomycin murine lung model miR-155 was upregulated and correlated with the degree of lung fibrosis [24]. Altogether, our observations reinforce the hypothesis of a strong immune activation in SSc-ILD with miR-155 as a key regulator.

Our observations that miR-21 is upregulated in the lungs of patients with SSc-ILD suggest that modulating miR-21 might be a future therapeutic strategy for SSc-ILD. miR-21 has been extensively studied in fibrosis, with increased expression observed in the lungs of bleomycin-treated mice and in the lungs of patients with idiopathic pulmonary fibrosis (IPF) in whom it is mainly localized in myofibroblasts [25]. More importantly, restoring miR-21 to normal levels inhibits bleomycin-induced pulmonary fibrosis [25]. miR-21 is also upregulated in the skin and fibroblasts from patients with both diffuse and limited subtypes of SSc [26]. miR-21 is induced by TGFβ in stellate hepatic cells and in a feed-forward loop it amplifies its signal, eventually promoting fibrosis [27]. Lung fibroblasts might not be the main cell type responsible for the miRNA signature in SSc-ILD, as we observed only a slight increase in miR-21 expression after TGFβ stimulation. Although our data on lung tissues in SSc-ILD do not permit a direct assessment of intracellular pathways, we showed that several upregulated profibrotic genes, such as Col3a1 and POSTN, correlate positively with miR-21 lung expression. In addition to being well-established as a profibrotic-miR, miR-21 is also central to many inflammatory pathways, and most importantly, in controlling the Toll-like receptor signaling with strong connection with miR-155 [28].

We also showed for the first time that miR-182 is upregulated in the lungs of patients with SSc-ILD. miR-182 has been implicated in several types of cancer and it is known to regulate proliferation, invasion, and migration of cancer cells [29]. Interestingly enough, some of the recently identified miR-182 targets are strong inhibitors of extracellular matrix degradation [30]. miR-4484 and miR-4459 were significantly downregulated in our cohort of patients with SSc-ILD and neither of them have validated target genes based on Tarbase v7.0 [13].

Our study has limitations. Our cohort was small and our results should ideally be validated in a larger group. The suggested mRNA-miRNA interactions and pathways are mainly predicted, although our agnostic and unbiased analysis using algorithmic tools guided us in similar directions, which support the validity of our results.

## Conclusions

Our unique approach of analyzing the relevance of miRNA in SSc-ILD using gene expression, pathway analysis, and functional studies, revealed a selected group of miRNA that might mediate the development and/or the progression of SSc-ILD. miR-155 and miR-21 were the most relevant miRNA having in common a close involvement in altered pathways observed in fibrotic diseases. mir-155 regulates the development of lung fibrosis in mice, thus, opening a new drug development opportunity.

## Abbreviations

%DLCO, percentage of diffusing capacity of the lungs for carbon monoxide; %FVC, percentage of forced vital capacity; ANOVA, analysis of variance; CCL18, chemokine C-C motif ligand 18; cDNA, complementary DNA; Col3a1, collagen type 3 alpha-1; Ct, cycle threshold; DIANA, DNA Intelligent Analysis; DMEM, Dulbecco’s modified Eagle’s medium; dSSc, diffuse systemic sclerosis; H&E, hematoxylin and eosin; FDR, false discovery rate; FiBMax, high-resolution computed tomography lung score; HRCT, high-resolution computed tomography; IFN, interferon alpha; IL-13, interleukin 13; KEGG, Kyoto Encyclopedia of Genes and Genomes; KO, knockout; lSSc, limited systemic sclerosis; mRNA, messenger RNA; miRNA, micro RNA; MS4A4A, membrane-spanning 4-domains, subfamily A, member 4A; NSIP, non-specific interstitial pneumonia; PBMC, peripheral blood mononuclear cells; PBS, phosphate-buffered saline; PFT, pulmonary function tests; POSTN, periostin; SPP1, osteopontin-1; SSc, systemic sclerosis; SSc-ILD, systemic sclerosis interstitial lung disease; TGF-beta, transforming growth factor beta; TIMP-1, tissue inhibitor of metalloproteinases type-1; WT, wild-type; IPF, idiopathic pulmonary fibrosis

## Additional files

Additional file 1: Table S1. The 185 differential miRNA expression (present in ≥25 % of samples, *q* < 0.25) in average fold-change observed in SSc-ILD compared to healthy controls. In sequence: transcript ID, fraction of samples. Top 30 upregulated and downregulated miRNA (present in >80 % of samples, *p* < 0.05) are highlighted in bold. (XLSX 55 kb) Additional file 2: Table S2. List of mRNA gene expression in SSc-ILD compared to healthy controls. In sequence: gene symbol, gene name, and average fold-change expression in SSc-ILD compared to healthy controls. (XLSX 15 kb) Additional file 3: Figure S1. List of the 57 KEGG pathways related to the most expressed miRNA in the lungs of patients with SSc-ILD compared to controls. In sequence: pathway names, significance levels based on *p* value, number of targeted genes, number of miRNA targeting each pathway. (PDF 1456 kb) Additional file 4: Table S3. Demographic data of patients with SSc-ILD and healthy controls for the miRNA analysis in PBMC. *%FVC* percentage forced vital capacity. *%DLCO* percentage diffusing capacity of the lung for carbon monoxide, *LSSc* limited systemic sclerosis, *DSSc* diffuse systemic sclerosis. (XLSX 35 kb) Additional file 5: Table S4. miRNA expression in PBMC by Nanostring analysis. Data are expressed as counts ofgenes and fold-change of the average on PBMC in SSc-ILD compared to the average of the controls (column U). (XLSX 167 kb) Additional file 6: Table S5. miRNA expression in lung fibroblasts by Nanostring analysis. Healthy control lung fibroblasts (HC) and SSc-ILD lung fibroblasts were stimulated with media, interleukin-13 (IL-13), interferon alpha (IFN), and TGF-beta for 18 hours. Data are expressed as counts of gene expression. (XLSX 56 kb) Additional file 7: Figure S2. miRNA expression of miR-193a (A) and miR-15b (B) on lung fibroblasts from cell lines of healthy controls (HC) and patients with SSc-ILD stimulated for 18 hours with media, IL-13, TGF-beta, and IFN-alpha. Data are expressed as fold-change compared to HC samples or media control. ANOVA, *p* > 0.05 for both miRNAs. (TIF 1112 kb) Additional file 8: Figure S3. Cluster of genes analyzed on lungs from wild-type and miR-155 KO mice exposed to PBS or bleomycin (see “Methods”). Two main clusters of genes upregulated (A) and downregulated (B) on lungs from wild-type mice exposed to bleomycin on day 28 of the model. Each miRNA was *z*-score-normalized across all samples and scaled to *red* and *blue* (≥2 or ≤ –2; respectively) and *white* indicating a *z*-score of zero. (TIF 3423 kb)
