# Supplementary material for: miR-155 in the progression of lung fibrosis in systemic sclerosis
Source: Arthritis Res Ther. 2016 Jul 5;18:155. doi: 10.1186/s13075-016-1054-6 (PMC4932708; doi:10.1186/s13075-016-1054-6)
Supplement: Additional file 3: Figure S1. — List of the 57 KEGG pathways related to the most expressed miRNA in the lungs of patients with SSc-ILD compared to controls. In sequence: pathway names, significance levels based on p value, number of targeted genes, number of miRNA targeting each pathway. (PDF 1456 kb) [file 13075_2016_1054_MOESM3_ESM.pdf]

#	KEGG pathway	p-value	#genes		#miRNAs	
1.	ECM-receptor interaction (hsa04512)	<1e-16	16	see genes	3	details
2.	Ubiquitin mediated proteolysis (hsa04120)	<1e-16	76	see genes	9	details
3.	Wnt signaling pathway (hsa04310)	<1e-16	79	see genes	10	details
4.	MAPK signaling pathway (hsa04010)	<1e-16	132	see genes	13	details
5.	Pathways in cancer (hsa05200)	<1e-16	166	see genes	15	details
6.	Axon guidance (hsa04360)	7.771561e-16	72	see genes	11	details
7.	PI3K-Akt signaling pathway (hsa04151)	2.742251e-14	137	see genes	12	details
8.	TGF-beta signaling pathway (hsa04350)	5.884182e-14	41	see genes	10	details
9.	ErbB signaling pathway (hsa04012)	1.172396e-13	40	see genes	12	details
10.	Transcriptional misregulation in cancer (hsa05202)	1.231237e-13	83	see genes	11	details
11.	Lysine degradation (hsa00310)	4.272138e-13	20	see genes	12	details
12.	Prostate cancer (hsa05215)	9.174883e-13	46	see genes	10	details
13.	Focal adhesion (hsa04510)	2.443379e-12	91	see genes	11	details
14.	Adherens junction (hsa04520)	6.371126e-12	43	see genes	9	details
15.	Neurotrophin signaling pathway (hsa04722)	1.918438e-10	66	see genes	13	details
16.	Endocytosis (hsa04144)	3.15572e-10	84	see genes	9	details
17.	Hepatitis B (hsa05161)	5.804531e-10	62	see genes	10	details
18.	Regulation of actin cytoskeleton (hsa04810)	6.585752e-10	98	see genes	10	details
19.	Glycosaminoglycan biosynthesis - chondroitin sulfate (hsa00532)	2.725444e-09	7	see genes	4	details
20.	Colorectal cancer (hsa05210)	7.339393e-09	34	see genes	9	details
21.	Renal cell carcinoma (hsa05211)	5.928736e-08	39	see genes	9	details
22.	Chronic myeloid leukemia (hsa05220)	6.408452e-08	35	see genes	6	details
23.	HTLV-I infection (hsa05166)	3.661482e-06	98	see genes	7	details
24.	Insulin signaling pathway (hsa04910)	0.0001957903	47	see genes	7	details
25.	Bacterial invasion of epithelial cells (hsa05100)	0.0002452291	33	see genes	7	details

25.	Bacterial invasion of epithelial cells (hsa05100)	0.0002452291	33	see genes	7	details
26.	Amoebiasis (hsa05146)	0.0002596306	27	see genes	4	details
27.	B cell receptor signaling pathway (hsa04662)	0.0003168009	31	see genes	6	details
28.	Vasopressin-regulated water reabsorption (hsa04962)	0.0008398865	11	see genes	3	details
29.	Acute myeloid leukemia (hsa05221)	0.0009800771	25	see genes	7	details
30.	Pancreatic cancer (hsa05212)	0.0009871219	30	see genes	5	details
31.	Basal cell carcinoma (hsa05217)	0.001585498	27	see genes	8	details
32.	Glioma (hsa05214)	0.001906011	23	see genes	7	details
33.	mRNA surveillance pathway (hsa03015)	0.004174632	37	see genes	2	details
34.	Small cell lung cancer (hsa05222)	0.00442103	37	see genes	8	details
35.	Endocrine and other factor-regulated calcium reabsorption (hsa04961)	0.00529026	23	see genes	6	details
36.	Endometrial cancer (hsa05213)	0.005680885	18	see genes	4	details
37.	Calcium signaling pathway (hsa04020)	0.006057219	25	see genes	3	details
38.	Gap junction (hsa04540)	0.007639207	26	see genes	4	details
39.	Cytokine-cytokine receptor interaction (hsa04060)	0.008086271	57	see genes	7	details
40.	T cell receptor signaling pathway (hsa04660)	0.008173359	36	see genes	6	details
41.	Dopaminergic synapse (hsa04728)	0.009865667	47	see genes	6	details
42.	HIF-1 signaling pathway (hsa04066)	0.01043302	45	see genes	6	details
43.	Melanoma (hsa05218)	0.01079892	32	see genes	6	details
44.	GnRH signaling pathway (hsa04912)	0.01086039	16	see genes	5	details
45.	Arrhythmogenic right ventricular cardiomyopathy (ARVC) (hsa05412)	0.01166638	27	see genes	3	details
46.	Glycosaminoglycan biosynthesis - heparan sulfate / heparin (hsa00534)	0.01242264	11	see genes	4	details
47.	Non-small cell lung cancer (hsa05223)	0.01322575	19	see genes	5	details
48.	Hypertrophic cardiomyopathy (HCM) (hsa05410)	0.01332318	30	see genes	6	details
49.	Aldosterone-regulated sodium reabsorption (hsa04960)	0.01842879	19	see genes	6	details
50.	Fc gamma R-mediated phagocytosis (hsa04666)	0.01901429	28	see genes	7	details
51.	mTOR signaling pathway (hsa04150)	0.01961992	24	see genes	7	details
52.	Gastric acid secretion (hsa04971)	0.02330469	27	see genes	7	details
53.	Circadian rhythm (hsa04710)	0.02455152	18	see genes	3	details
54.	Long-term potentiation (hsa04720)	0.04191049	30	see genes	5	details
55.	Hedgehog signaling pathway (hsa04340)	0.04343957	21	see genes	5	details
56.	Dilated cardiomyopathy (hsa05414)	0.04441793	14	see genes	4	details
57.	Protein processing in endoplasmic reticulum (hsa04141)	0.04525695	51	see genes	2	details
