# Supplementary figures and images for: miR-155 in the progression of lung fibrosis in systemic sclerosis
Source: Arthritis Res Ther. 2016 Jul 5;18:155. doi: 10.1186/s13075-016-1054-6 (PMC4932708; doi:10.1186/s13075-016-1054-6)

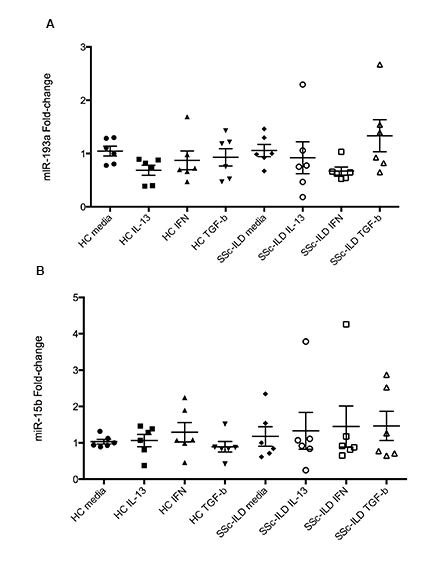

Supplement: Additional file 7: Figure S2. — miRNA expression of miR-193a (A) and miR-15b (B) on lung fibroblasts from cell lines of healthy controls (HC) and patients with SSc-ILD stimulated for 18 hours with media, IL-13, TGF-beta, and IFN-alpha. Data are expressed as fold-change compared to HC samples or media control. ANOVA, p > 0.05 for both miRNAs. (TIF 1112 kb) [file 13075_2016_1054_MOESM7_ESM.tif]

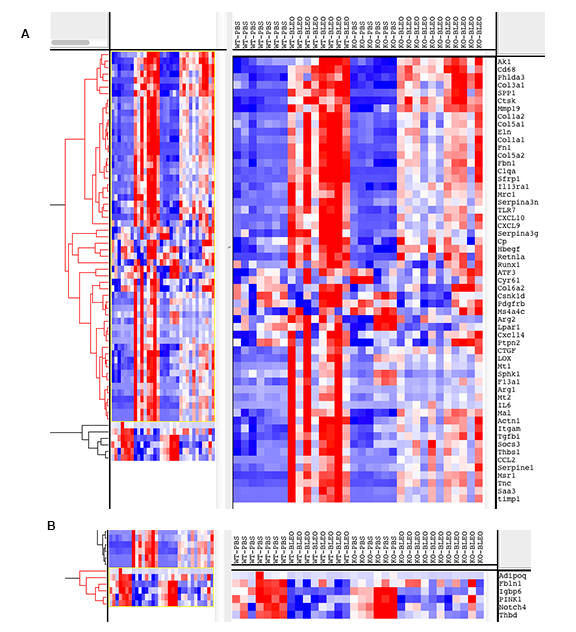

Supplement: Additional file 8: Figure S3. — Cluster of genes analyzed on lungs from wild-type and miR-155 KO mice exposed to PBS or bleomycin (see “Methods”). Two main clusters of genes upregulated (A) and downregulated (B) on lungs from wild-type mice exposed to bleomycin on day 28 of the model. Each miRNA was z-score-normalized across all samples and scaled to red and blue (≥2 or ≤ –2; respectively) and white indicating a z-score of zero. (TIF 3423 kb) [file 13075_2016_1054_MOESM8_ESM.tif]
